# Pulmonary hypertension in a hemodialysis porcine model: possible unforeseen causes

**DOI:** 10.1080/0886022X.2022.2045204

**Published:** 2022-06-07

**Authors:** Karim M. Soliman, Vinaya Rao, Ahmed Daoud

**Affiliations:** a Department of Medicine, Division of Nephrology, Medical University of South Carolina, Charleston, SC, USA; b Department of Surgery, Division of Transplant Surgery, Medical University of South Carolina, Charleston, SC, USA; c Department of Medicine, Division of Nephrology, Cairo University, Giza, Egypt

Dear Editor,

We have read with great interest the manuscript written by Pethő et al. about hemodialyzer reactions [1]. The authors found that hemodialysis was associated with increase in pulmonary artery pressure (PAP) and pulmonary hypertension (PAH) in pigs. The increase in PAP was gradual during dialysis and sudden after blood reinfusion. The cause of this increase in PAP remains unclear. We would assume, as customary in human dialysis, normal sodium-chloride (0.9%) is utilized to rinse back the extracorporeal circulating volume, with volume of 300–500 mL.

While the concept of PAH was thoroughly discussed, we would like to add our thoughts by shedding light on some unforeseen causes and hypotheses of PAH. Normal saline is known to be acidic with a pH of 5.5, and reported as low as 4.6 [2,3]. Pulmonary vasoconstriction has been linked to acidosis, which can lead to PAH [4]. As a result, rinsing with NS is an unanticipated cause of PAH. Despite the small amount of saline typically used to rinse the dialyzer, it is assumed to be enough to lower the pH in a pig with a smaller blood volume. Another theory is that hemodialysis-induced complement activation produces pulmonary vasoconstriction and, as a result, leading to PAH [5]. Additionally, the likelihood that the dialyzer is removing certain ‘unknown’ vasodilator substances by binding to the dialyzer membrane should be seriously considered and further investigated. Finally, it is tempting to speculate that some these ‘unknown’ substances get eluded, perhaps as the function of lower pH, with the saline-containing rinse-back of the extracorporeal circulating blood volume. If this is the case, pulmonary vasoconstriction and PAH may result.
